# 3-[(*E*)-4-Methoxy­benzyl­idene]-1-methyl­piperidin-4-one

**DOI:** 10.1107/S1600536808003280

**Published:** 2008-02-06

**Authors:** D. Gayathri, D. Velmurugan, R. Ranjith Kumar, S. Perumal, K. Ravikumar

**Affiliations:** a Centre of Advanced Study in Crystallography and Biophysics, University of Madras, Guindy Campus, Chennai 600 025, India; b Department of Organic Chemistry, School of Chemistry, Madurai Kamaraj University, Madurai 625 021, India; c Laboratory of X-ray Crystallography, Indian Institute of Chemical Technology, Hyderabad 500 007, India

## Abstract

The piperidone ring of the title compound, C_14_H_17_NO_2_, adopts a half-chair conformation. The crystal packing is stabilized by inter­molecular C—H⋯O inter­actions, which generate a *C*(8) chain running along the *b* axis.

## Related literature

For related literature, see: Abignente & Biniecka-Picazio (1977 ▶); Angle & Breitenbucher (1995 ▶); Cremer & Pople (1975 ▶); Nardelli (1983 ▶); Wang & Wuorola (1992 ▶).
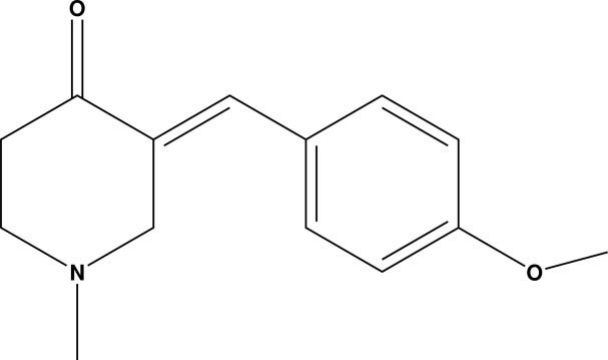


## Experimental

### 

#### Crystal data

C_14_H_17_NO_2_
*M* *_r_* = 231.29
Orthorhombic, 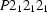

*a* = 7.5212 (7) Å
*b* = 12.4097 (11) Å
*c* = 13.5062 (12) Å
*V* = 1260.6 (2) Å^3^
*Z* = 4
Mo *K*α radiation
μ = 0.08 mm^−1^
*T* = 293 (2) K
0.24 × 0.22 × 0.21 mm

#### Data collection

Bruker SMART CCD area-detector diffractometer
Absorption correction: none
10874 measured reflections
1729 independent reflections
1577 reflections with *I* > 2σ(*I*)
*R*_int_ = 0.019

#### Refinement

*R*[*F* ^2^ > 2σ(*F* ^2^)] = 0.044
*wR*(*F* ^2^) = 0.128
*S* = 1.11
1729 reflections
156 parameters
H-atom parameters constrained
Δρ_max_ = 0.28 e Å^−3^
Δρ_min_ = −0.13 e Å^−3^

### 

Data collection: *SMART* (Bruker, 2001 ▶); cell refinement: *SAINT* (Bruker, 2001 ▶); data reduction: *SAINT*; program(s) used to solve structure: *SHELXS97* (Sheldrick, 2008 ▶); program(s) used to refine structure: *SHELXL97* (Sheldrick, 2008 ▶); molecular graphics: *PLATON* (Spek, 2003 ▶); software used to prepare material for publication: *SHELXL97* and *PARST* (Nardelli, 1995 ▶).

## Supplementary Material

Crystal structure: contains datablocks I, global. DOI: 10.1107/S1600536808003280/bt2673sup1.cif

Structure factors: contains datablocks I. DOI: 10.1107/S1600536808003280/bt2673Isup2.hkl

Additional supplementary materials:  crystallographic information; 3D view; checkCIF report

## Figures and Tables

**Table 1:** Hydrogen-bond geometry (Å, °)

*D*—H⋯*A*	*D*—H	H⋯*A*	*D*⋯*A*	*D*—H⋯*A*
C10—H10⋯O1^i^	0.93	2.54	3.419 (3)	157

Symmetry code: (i) 
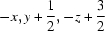
.

**Fig. 1.**
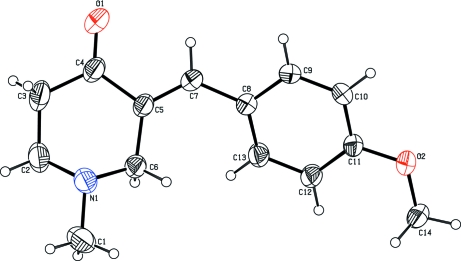
The molecular structure of title compound, showing 30% probability displacement ellipsoids.

**Fig. 2.**
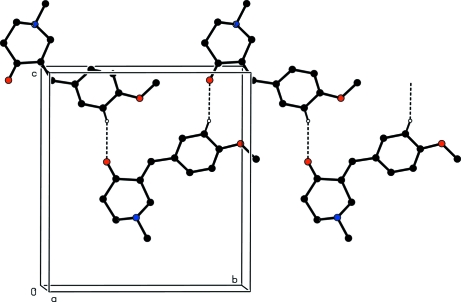
The molecular packing of the title compound, viewed down the a axis.

## supplementary crystallographic information

### Comment

Substituted 4-piperidones are important synthetic intermediates for the preparation of various pharmaceuticals (Wang & Wuorola, 1992). 4-Piperidones are also widely prevalent in natural products such as alkaloids (Angle & Breitenbucher, 1995). Derivatives of 4-piperidones have been found to exhibit spasmolytic activities (Abignente & Biniecka-Picazio, 1977). Since, the title compound is pharmacologically important, the crystal structure of the title compound has been determined by X-ray diffraction.

The sum of the bond angles around N1 [331.4 (6)°] indicate the *sp*^3^ hybridization. The torsion angles around C10—C11—O2—C14 [179.4 (2)°] and C12—C11—O2—C14 [-0.8 (3)°] indicate that the methoxy group is planar with the phenyl ring.

The piperidone ring adopts a half-chair conformation with the puckering parameters (Cremer & Pople, 1975) and the smallest displacement asymmetry parameters (Nardelli, 1983) being q_2_ = 0.366 (3) Å, q_3_ = 0.361 (3) Å, Q_T_ = 0.515 (3)Å and θ = 45.4 (3)°. The molecular conformation is stabilized by weak C—H···O intramolecular interactions. The crystal packing is stabilized by C—H···O intermolecular interactions generating a chain C(8) running along *b* axis.

### Experimental

A mixture of 1-methyl-4-piperidone (1 mmol) and pyrrolidine (1.2 mmol) was taken in a glass tube, mixed well and kept aside for 5 min at ambient temperature. To this mixture, 4-methoxybenzaldehyde (1 mmol) was added, mixed thoroughly and the tube containing the mixture was partially immersed in a silica bath placed in a microwave oven and irradiated at 4 power level for 8 minutes. The progress of the reaction was monitored after every 1 min of irradiation by TLC with petroleum ether:ethyl acetate (1:2 *v*/*v* mixture) as eluent. After each irradiation, the reaction mixture was cooled to room temperature and mixed well. The maximum temperature of the silica bath, measured immediately after each irradiation was over by stirring the silica bath with the thermometer, was found to be 65 °C. After completion of the reaction as evident from the TLC, the product was purified by column chromatography using petroleum ether:ethyl acetate (7:2 *v*/*v*) mixture and crystallized from ethyl acetate.

### Refinement

In the absence of anomalous scatterers Friedel pairs had been merged prior to refinement. All H-atoms were refined using a riding model with d(C—H) = 0.93 Å, *U*_iso_=1.2*U*_eq_ (C) for aromatic C atoms, 0.97 Å, *U*_iso_ = 1.2*U*_eq_ (C) for methylene and 0.96 Å, *U*_iso_ = 1.5*U*_eq_ (C) for methyl groups.

### Crystal data

**Table:**

C_14_H_17_NO_2_	*F*_000_ = 496
*M**_r_* = 231.29	*D*_x_ = 1.219 Mg m^−^^3^
Orthorhombic, *P*2_1_2_1_2_1_	Mo *K*α radiation λ = 0.71073 Å
Hall symbol: P 2ac 2ab	Cell parameters from 861 reflections
*a* = 7.5212 (7) Å	θ = 2.2–25.0º
*b* = 12.4097 (11) Å	µ = 0.08 mm^−^^1^
*c* = 13.5062 (12) Å	*T* = 293 (2) K
*V* = 1260.6 (2) Å^3^	Block, pale yellow
*Z* = 4	0.24 × 0.22 × 0.21 mm

### Data collection

**Table:**

Bruker SMART CCD area-detector diffractometer	1577 reflections with *I* > 2σ(*I*)
Radiation source: fine-focus sealed tube	*R*_int_ = 0.019
Monochromator: graphite	θ_max_ = 28.1º
*T* = 293(2) K	θ_min_ = 2.2º
ω scans	*h* = −9→9
Absorption correction: none	*k* = −16→16
10874 measured reflections	*l* = −17→17
1729 independent reflections	

### Refinement

**Table:**

Refinement on *F*^2^	Hydrogen site location: inferred from neighbouring sites
Least-squares matrix: full	H-atom parameters constrained
*R*[*F*^2^ > 2σ(*F*^2^)] = 0.045	*w* = 1/[σ^2^(*F*_o_^2^) + (0.083*P*)^2^ + 0.0611*P*] where *P* = (*F*_o_^2^ + 2*F*_c_^2^)/3
*wR*(*F*^2^) = 0.128	(Δ/σ)_max_ < 0.001
*S* = 1.11	Δρ_max_ = 0.28 e Å^−^^3^
1729 reflections	Δρ_min_ = −0.13 e Å^−^^3^
156 parameters	Extinction correction: none
Primary atom site location: structure-invariant direct methods	Absolute structure:
Secondary atom site location: difference Fourier map	

### Special details

**Table:**

Geometry. All e.s.d.'s (except the e.s.d. in the dihedral angle between two l.s. planes) are estimated using the full covariance matrix. The cell e.s.d.'s are taken into account individually in the estimation of e.s.d.'s in distances, angles and torsion angles; correlations between e.s.d.'s in cell parameters are only used when they are defined by crystal symmetry. An approximate (isotropic) treatment of cell e.s.d.'s is used for estimating e.s.d.'s involving l.s. planes.
Refinement. Refinement of *F*^2^ against ALL reflections. The weighted *R*-factor *wR* and goodness of fit *S* are based on *F*^2^, conventional *R*-factors *R* are based on *F*, with *F* set to zero for negative *F*^2^. The threshold expression of *F*^2^ > σ(*F*^2^) is used only for calculating *R*-factors(gt) *etc*. and is not relevant to the choice of reflections for refinement. *R*-factors based on *F*^2^ are statistically about twice as large as those based on *F*, and *R*- factors based on ALL data will be even larger.

### Fractional atomic coordinates and isotropic or equivalent isotropic displacement parameters (Å^2^)

**Table:**

	*x*	*y*	*z*	*U*_iso_*/*U*_eq_	
C1	−0.1137 (5)	0.0252 (2)	1.28219 (18)	0.0864 (8)	
H1A	−0.0039	0.0433	1.3144	0.130*	
H1B	−0.1816	−0.0218	1.3241	0.130*	
H1C	−0.1803	0.0898	1.2697	0.130*	
C2	0.0338 (4)	−0.1243 (2)	1.20225 (17)	0.0802 (7)	
H2A	−0.0149	−0.1685	1.2549	0.096*	
H2B	0.1533	−0.1031	1.2210	0.096*	
C3	0.0400 (5)	−0.18853 (18)	1.1072 (2)	0.0845 (7)	
H3A	0.1321	−0.2429	1.1131	0.101*	
H3B	−0.0725	−0.2257	1.0992	0.101*	
C4	0.0749 (4)	−0.12308 (16)	1.01586 (16)	0.0697 (6)	
C5	0.0494 (3)	−0.00418 (14)	1.02172 (14)	0.0548 (5)	
C6	0.0120 (3)	0.04524 (15)	1.12177 (14)	0.0558 (4)	
H6A	0.1232	0.0685	1.1512	0.067*	
H6B	−0.0622	0.1085	1.1130	0.067*	
C7	0.0594 (3)	0.05043 (14)	0.93680 (15)	0.0560 (5)	
H7	0.0790	0.0090	0.8805	0.067*	
C8	0.0436 (3)	0.16662 (14)	0.91996 (13)	0.0510 (4)	
C9	−0.0213 (3)	0.20203 (14)	0.82852 (13)	0.0562 (5)	
H9	−0.0506	0.1515	0.7804	0.067*	
C10	−0.0427 (3)	0.30978 (15)	0.80817 (12)	0.0576 (5)	
H10	−0.0879	0.3314	0.7472	0.069*	
C11	0.0031 (2)	0.38597 (14)	0.87840 (13)	0.0511 (4)	
C12	0.0740 (3)	0.35353 (14)	0.96828 (14)	0.0585 (5)	
H12	0.1079	0.4045	1.0151	0.070*	
C13	0.0938 (3)	0.24434 (17)	0.98781 (15)	0.0581 (5)	
H13	0.1422	0.2229	1.0481	0.070*	
C14	0.0194 (4)	0.57070 (15)	0.92477 (17)	0.0667 (6)	
H14A	−0.0449	0.5570	0.9849	0.100*	
H14B	−0.0116	0.6407	0.8999	0.100*	
H14C	0.1448	0.5681	0.9379	0.100*	
N1	−0.0762 (3)	−0.02883 (15)	1.18885 (13)	0.0656 (5)	
O1	0.1230 (4)	−0.16737 (12)	0.94008 (14)	0.0993 (7)	
O2	−0.0251 (2)	0.49113 (10)	0.85323 (10)	0.0627 (4)	

### Atomic displacement parameters (Å^2^)

**Table:**

	*U*^11^	*U*^22^	*U*^33^	*U*^12^	*U*^13^	*U*^23^
C1	0.105 (2)	0.0972 (18)	0.0569 (12)	−0.0171 (16)	0.0036 (13)	0.0011 (12)
C2	0.1040 (18)	0.0634 (11)	0.0731 (13)	−0.0072 (14)	−0.0128 (14)	0.0222 (11)
C3	0.1134 (19)	0.0459 (9)	0.0942 (16)	−0.0027 (13)	−0.0080 (17)	0.0165 (11)
C4	0.0965 (15)	0.0404 (8)	0.0723 (12)	0.0031 (10)	−0.0171 (13)	−0.0023 (9)
C5	0.0662 (11)	0.0391 (7)	0.0590 (10)	0.0011 (8)	−0.0092 (9)	−0.0002 (7)
C6	0.0660 (11)	0.0464 (8)	0.0551 (9)	−0.0030 (8)	−0.0093 (9)	0.0009 (7)
C7	0.0701 (11)	0.0430 (8)	0.0551 (9)	0.0031 (8)	−0.0019 (9)	−0.0053 (7)
C8	0.0603 (10)	0.0433 (8)	0.0493 (8)	0.0005 (8)	0.0021 (8)	0.0001 (6)
C9	0.0787 (12)	0.0476 (8)	0.0423 (7)	−0.0009 (9)	0.0058 (9)	−0.0040 (6)
C10	0.0793 (12)	0.0538 (9)	0.0397 (7)	0.0015 (10)	0.0022 (9)	0.0058 (7)
C11	0.0602 (9)	0.0421 (7)	0.0510 (8)	−0.0017 (8)	0.0055 (8)	0.0055 (7)
C12	0.0744 (11)	0.0443 (9)	0.0568 (10)	−0.0085 (9)	−0.0103 (9)	−0.0004 (7)
C13	0.0713 (11)	0.0488 (9)	0.0544 (9)	−0.0029 (9)	−0.0150 (9)	0.0057 (7)
C14	0.0878 (14)	0.0414 (8)	0.0708 (12)	−0.0001 (10)	0.0100 (12)	−0.0005 (8)
N1	0.0784 (11)	0.0618 (9)	0.0565 (8)	−0.0120 (9)	−0.0084 (9)	0.0061 (7)
O1	0.169 (2)	0.0465 (7)	0.0828 (11)	0.0139 (12)	−0.0067 (13)	−0.0083 (8)
O2	0.0866 (10)	0.0415 (6)	0.0600 (7)	−0.0016 (7)	−0.0008 (8)	0.0077 (5)

### Geometric parameters (Å, °)

**Table:**

C1—N1	1.456 (3)	C7—C8	1.465 (2)
C1—H1A	0.9600	C7—H7	0.9300
C1—H1B	0.9600	C8—C13	1.383 (3)
C1—H1C	0.9600	C8—C9	1.399 (3)
C2—N1	1.457 (3)	C9—C10	1.374 (3)
C2—C3	1.511 (4)	C9—H9	0.9300
C2—H2A	0.9700	C10—C11	1.383 (3)
C2—H2B	0.9700	C10—H10	0.9300
C3—C4	1.500 (3)	C11—O2	1.365 (2)
C3—H3A	0.9700	C11—C12	1.385 (3)
C3—H3B	0.9700	C12—C13	1.389 (3)
C4—O1	1.217 (3)	C12—H12	0.9300
C4—C5	1.490 (3)	C13—H13	0.9300
C5—C7	1.334 (3)	C14—O2	1.421 (3)
C5—C6	1.510 (3)	C14—H14A	0.9600
C6—N1	1.451 (3)	C14—H14B	0.9600
C6—H6A	0.9700	C14—H14C	0.9600
C6—H6B	0.9700		
			
N1—C1—H1A	109.5	C5—C7—H7	115.5
N1—C1—H1B	109.5	C8—C7—H7	115.5
H1A—C1—H1B	109.5	C13—C8—C9	117.47 (16)
N1—C1—H1C	109.5	C13—C8—C7	124.17 (18)
H1A—C1—H1C	109.5	C9—C8—C7	118.33 (16)
H1B—C1—H1C	109.5	C10—C9—C8	121.53 (17)
N1—C2—C3	109.93 (19)	C10—C9—H9	119.2
N1—C2—H2A	109.7	C8—C9—H9	119.2
C3—C2—H2A	109.7	C9—C10—C11	119.92 (17)
N1—C2—H2B	109.7	C9—C10—H10	120.0
C3—C2—H2B	109.7	C11—C10—H10	120.0
H2A—C2—H2B	108.2	O2—C11—C12	123.75 (16)
C4—C3—C2	114.74 (18)	O2—C11—C10	116.37 (16)
C4—C3—H3A	108.6	C12—C11—C10	119.88 (16)
C2—C3—H3A	108.6	C11—C12—C13	119.43 (17)
C4—C3—H3B	108.6	C11—C12—H12	120.3
C2—C3—H3B	108.6	C13—C12—H12	120.3
H3A—C3—H3B	107.6	C8—C13—C12	121.69 (18)
O1—C4—C5	122.0 (2)	C8—C13—H13	119.2
O1—C4—C3	119.95 (19)	C12—C13—H13	119.2
C5—C4—C3	118.0 (2)	O2—C14—H14A	109.5
C7—C5—C4	116.75 (17)	O2—C14—H14B	109.5
C7—C5—C6	124.98 (16)	H14A—C14—H14B	109.5
C4—C5—C6	118.27 (16)	O2—C14—H14C	109.5
N1—C6—C5	112.74 (16)	H14A—C14—H14C	109.5
N1—C6—H6A	109.0	H14B—C14—H14C	109.5
C5—C6—H6A	109.0	C6—N1—C1	109.72 (18)
N1—C6—H6B	109.0	C6—N1—C2	109.5 (2)
C5—C6—H6B	109.0	C1—N1—C2	112.18 (19)
H6A—C6—H6B	107.8	C11—O2—C14	117.27 (15)
C5—C7—C8	128.98 (17)		
			
N1—C2—C3—C4	46.7 (3)	C8—C9—C10—C11	1.1 (3)
C2—C3—C4—O1	163.0 (3)	C9—C10—C11—O2	−178.8 (2)
C2—C3—C4—C5	−16.8 (4)	C9—C10—C11—C12	1.3 (3)
O1—C4—C5—C7	8.4 (4)	O2—C11—C12—C13	178.5 (2)
C3—C4—C5—C7	−171.8 (2)	C10—C11—C12—C13	−1.7 (3)
O1—C4—C5—C6	−172.5 (3)	C9—C8—C13—C12	2.7 (3)
C3—C4—C5—C6	7.3 (3)	C7—C8—C13—C12	−179.2 (2)
C7—C5—C6—N1	151.3 (2)	C11—C12—C13—C8	−0.4 (3)
C4—C5—C6—N1	−27.8 (3)	C5—C6—N1—C1	−178.0 (2)
C4—C5—C7—C8	−178.6 (2)	C5—C6—N1—C2	58.5 (2)
C6—C5—C7—C8	2.3 (4)	C3—C2—N1—C6	−68.5 (3)
C5—C7—C8—C13	30.6 (4)	C3—C2—N1—C1	169.4 (2)
C5—C7—C8—C9	−151.3 (2)	C12—C11—O2—C14	−0.8 (3)
C13—C8—C9—C10	−3.1 (3)	C10—C11—O2—C14	179.37 (19)
C7—C8—C9—C10	178.8 (2)		

### Hydrogen-bond geometry (Å, °)

**Table:**

*D*—H···*A*	*D*—H	H···*A*	*D*···*A*	*D*—H···*A*
C10—H10···O1^i^	0.93	2.54	3.419 (3)	157

Symmetry codes: (i) −*x*, *y*+1/2, −*z*+3/2.
